# The immunomodulatory effect of IL-4 accelerates bone substitute material-mediated osteogenesis in aged rats via NLRP3 inflammasome inhibition

**DOI:** 10.3389/fimmu.2023.1121549

**Published:** 2023-04-20

**Authors:** Duchenhui Li, Xiao Li, Jie Zhang, Zhenglong Tang, Ai Tian

**Affiliations:** ^1^ Department of Prosthodontics and Implantology, School and Hospital of Stomatology of Guizhou Medical University, Guiyang, Guizhou, China; ^2^ Department of Oral and Maxillofacial Surgery, School and Hospital of Stomatology of Guizhou Medical University, Guiyang, China; ^3^ Department of Physiology and Pathology, School of Basic Medical Sciences, Guizhou Medical University, Guiyang, China; ^4^ Department of Oral and Maxillofacial Surgery, Guiyang Hospital of Stomatology, Guiyang, Guizhou, China

**Keywords:** immunosenescence, immunomodulation, osteogenesis, macrophages, bone substitutes materials

## Abstract

**Background:**

Bone defect repair by implanting bone substitute materials has been a common clinical treatment. With the understanding of substance–immune system interactions and increasing evidence indicating that the post-implantation immune response determines the fate of bone substitute materials, active modulation of host macrophage polarization is considered a promising strategy. However, whether the same regulatory effects exist when an individual immune system is altered with aging is unclear.

**Methods:**

In this study, we mechanistically investigated the effect of immunosenescence on the active regulation of macrophage polarization by establishing a cranial bone defect model in young and aged rats implanted with Bio-Oss®. Forty-eight young and 48 aged specific pathogen-free (SPF) male SD rats were randomly divided into two groups. In the experimental group, 20 μL of IL-4 (0.5 μg/mL) was injected locally on the third to seventh postoperative days, while an equal volume of PBS was injected in the control group. Specimens were collected at 1, 2, 6, and 12 weeks postoperatively, and bone regeneration at the defect site was evaluated by micro-CT, histomorphometry, immunohistochemistry, double-labeling immunofluorescence, and RT–qPCR.

**Results:**

The application of exogenous IL-4 reduced activation of NLRP3 inflammasomes by promoting the polarization of M1 macrophages to M2 macrophages, thus promoting bone regeneration at the site of bone defects in aged rats. However, this effect was gradually weakened after the IL-4 intervention was discontinued.

**Conclusion:**

Our data confirmed that a strategy to regulate macrophage polarization is also feasible under conditions of immunosenescence, i.e., the local inflammatory microenvironment can be regulated by reducing M1-type macrophages. However, further experiments are needed to determine an exogenous IL-4 intervention that can maintain a more sustained effect.

## 1 Introduction

Bone defects caused by trauma, cancer, or skeletal diseases bring great pain to patients both physically and psychologically, and represent a major clinical problem worldwide (1). At present, bone defect repair methods are roughly divided into three categories, including autografts, allografts, and bone substitute materials grafts (2). In related fields, researchers have attempted to obtain more suitable bone substitute materials by optimizing the chemical composition, three-dimensional structure, and mechanical properties of bone substitute materials (3). However, in recent years, increasing evidence has demonstrated that the interaction between bone substitute materials and the immune system is a key factor affecting bone regeneration and that the host’s immune response after bone substitute material implantation determines the success rate of bone regeneration (4, 5).

Macrophages can direct host inflammatory and immune processes and play an important role in tissue repair and regeneration. Bone macrophages present in bone tissue control the bone remodeling process by regulating the balance between osteoblasts and osteoclasts (6). Macrophages originate from monocytes in the bone marrow and are an important component of the innate immune system (7). On the basis of surface molecule expression, macrophages can be divided into various subtypes, including M1 and M2 (8). By secreting a variety of cytokines (IL-6, TNF-a, etc.), M1 macrophages recruit immune cells and bone marrow stromal stem cells in the circulatory system to clear damaged tissue and maintain tissue homeostasis (6). M2 macrophages promote tissue regeneration and repair by secreting anti-inflammatory factors (such as IL-4 and IL-10), recruiting mesenchymal stem cells, and producing growth factors that regulate cell differentiation (such as BMP-2 and VEGF) (9). During tooth movement in mice, an increase in the M1/M2 ratio exacerbates root resorption, whereas a decrease in the M1/M2 ratio partially rescues root resorption (10), indicating that the M1/M2 ratio is critical for bone tissue regeneration (11). Existing studies have shown that IL-4-induced M2 macrophages enhance alkaline phosphatase (ALP) activity and osteocalcin (OCN) secretion in osteoblasts and promote bone mineralization, thereby enhancing bone regeneration (12). The sustained delivery of an appropriate amount of IL-4 can achieve an ideal M1/M2 macrophage ratio, form a local microenvironment conducive to healing, and enhance the osteogenic effect induced by local bone substitute materials (13). Numerous *in vivo* and *in vitro* experiments have demonstrated the feasibility of promoting the osteogenesis of bone substitute materials by regulating macrophage polarization (14, 15).

However, the incidence of bone defects has increased as the elderly population has increased, and these individuals have become a key population in which bone substitute materials are used. With increasing age, immune function (including adaptability and the innate immune system) gradually declines, referred to as immunosenescence (16). Its main feature is persistent chronic mild inflammation, also known as inflammatory aging (17). Due to the inflammatory microenvironment, elderly individuals may have a compensatory regulatory mechanism, and overcompensation may lead to immune disorders in the aging process, thereby delaying repair bone defect and further affecting the use of modulatory drugs that target immune cell subpopulations (including macrophages) (18). Löffler et al. (19) found that the overall expression of the monocyte/pan-macrophage markers CD14 and CD68 was reduced in a fracture model of aged rats and that the expression of anti-inflammatory M2 macrophage markers was low in hematomas of elderly animals. Gibon et al. (20) showed that the polarization of mouse bone marrow-derived macrophages changes with age; a large number of iNOS/CD206 double-positive M0 macrophages were observed in elderly individuals, and Arg1/CD206 mRNA expression was absent in M2 macrophages. Macrophages involved in the process of bone healing and inflammation show age correlations, with impaired cytokine secretion and polarization with increases in age (20).

NLRP3 inflammasomes are major innate immune sensors (21) that mediate age-related inflammation through the recognition of endogenous damage-related molecular patterns (DAMPs) during the aging process (22). After activation, NLRP3 inflammasomes activate caspase-1, which in turn cleaves pro-IL-1β to form mature IL-1β (23). IL-1β promotes receptor activation of nuclear factor kappa-B ligand (RANKL) and induces osteoclast differentiation (24). Recent research evidence indicates that in periodontal tissue, NLRP3 inflammasomes expressed by macrophages are associated with alveolar bone degeneration caused by aging in mice and that inhibition of NLRP3 inflammasomes significantly reduces alveolar bone resorption in aged mice (25). However, the effect of immunosenescence on regulating macrophage polarization as a strategy for bone tissue regeneration is currently unknown.

The inflammatory response is an important stage of bone injury repair, and the altered macrophage phenotype and local inflammatory microenvironment accompanying immunosenescence are closely related to the delayed repair of bone injury in the elderly population (18–20). Although IL-4 can promote the osteogenic effect of bone substitute materials, whether there are differences in elderly individuals has not been reported. Therefore, we speculated that the local inflammatory microenvironment could be altered by modulating the direction of macrophage polarization to reduce inflammatory senescence and improve osteogenesis outcomes after transplantation of bone substitute materials in the elderly population. In this study, a rat cranial bone defect model was established and implanted with Bio-Oss®. The classic M2-polarizing cytokine IL-4 was delivered into the defect region. Then, we investigated differences in the polarization phenotypes of macrophages and the effects of osteogenic/osteoblastic protein expression on bone regeneration mediated by bone substitute materials after IL-4 intervention in young and aged rats. The effects of local inflammatory factor expression (NLRP3-related molecules cl-caspase-1 and IL-1β) and IL-4-mediated macrophage polarization on bone regeneration with bone substitute materials under immunosenescence conditions were examined in aged rats.

## 2 Materials and methods

### 2.1 Animals and *in vivo* studies

**Figure 1:**
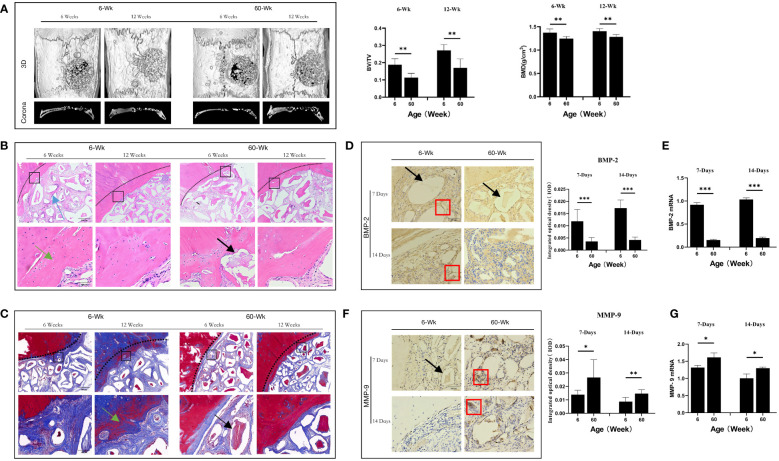
**(A)** Three-dimensional and coronal micro-CT images of the cranial bone defect, the bone volume/total volume fraction (BV/TV), and bone mineral density (BMD) at 6 and 12 weeks postoperatively. Bone repair was weaker in the elderly group than in the young group. **(B)** H&E and **(C)** Masson’s trichrome staining further confirmed the micro-CT results indicating that bone repair was weaker in the elderly group than in the young group. Green arrows: new bone tissue; Black arrows: demineralized Bio-Oss® materials; Blue arrows: bone island. **(D, F)** Immunohistochemical detection of BMP-2 and MMP-9 expression in Bio-Oss® and surrounding tissues on day 7 and day 14; Red square: positive cells; Black arrows: demineralized Bio-Oss® materials. **(E, G)** Local BMP-2 and MMP-9 gene expression in young and aged SD rats reflecting the dynamic balance of bone regeneration and bone resorption in young and aged rats with local defects. The osteogenic effect of the bone substitute material was significantly reduced in aged rats. *P<0.05, **P<0.01, ***P<0.001.

Sprague Dawley (SD) rats were obtained from the Animal Experimental Center of Guizhou Medical University. All SD rats were housed in accordance with the *Guide for the Care and Use of Laboratory Animals* by the National Institute of Health. The study protocol was approved by the Ethics Committee of Guizhou Medical University. Forty-eight 6-week-old (200 g ± 20 g) male SD rats were randomly divided into two groups: young control group (n=24) and young treatment group (n=24). Forty-eight 60-week-old (500 g ± 50 g) male SD rats were randomly divided into two groups: elderly control group (n=24) and elderly treatment group (n=24). The cranial bone defect model was used in this study as the most rapid, stable, and intuitive *in vivo* assessment method for bone regeneration (26). Briefly, SD rats underwent intraperitoneal anesthesia with 5% pentobarbital (35 mg/kg) and subcutaneous injection of 2% lidocaine containing 1:100,000 epinephrine in the operative area. A 1.5-2-cm sagittal incision was made on the top of the cranial bone. The periosteum was stripped to expose the cranial bone, and then a critical bone defect of 5 mm was created by a trephine with inner diameter 5 mm, which was continuously cooled with sterile saline. Bio-Oss^®^ was implanted into the bone defect. The periosteum and skin were sutured in layers (**Figure 1A**).

From the third to seventh days after surgery, the animals were anesthetized, and rats in the treatment group were injected (into the implantation area) with recombinant IL-4 (0.5 µg/ml, Peprotech, USA) once daily; the volume of each injection was 20 μL (13). The control group was injected with the same volume of phosphate buffered saline (PBS) (Peprotech, USA).

Bone substitute materials and surrounding bone tissue were collected for histological (immunohistochemistry and immunofluorescence) and RT-qPCR analyses at 7 days, 14 days, 6 weeks and 12 weeks after surgery. Serum was collected for enzyme-linked immunosorbent assays (ELISAs).

### 2.2 Micro-computed tomography (CT) analysis

Micro-CT scanning and imaging were performed on a Nemo Micro-CT (NMC-100) machine (PINGSENG Health care Inc.). The rat skull was placed horizontally in the sample chamber. The sample was kept still during the scanning process. The detection ring was rotated 360° around the horizontal axis of the sample chamber, and circular projections were performed 4000 times per circumference, scanning a total of three circumferential positions. After scanning, 3D images were reconstructed using the FDK method in Avatar (version 1.6.2, PINGSENG Health care Inc.), and the bone volume fraction (BV/TV, %) and bone mass density (BMD, g/cm^3^) were quantitatively analyzed.

A cylindrical region (diameter of 5 mm and thickness of 1 mm) was selected as the region of interest (ROI), and the trabecular bone, implant, and cortical bone in the ROI were segmented through image processing methods such as threshold segmentation and region growth. The total volume of trabecular bone and cortical bone was used as the BV value to calculate the bone analysis parameters, such as BV/TV, thickness, and number. In addition, the BMD value of the bone fraction in the ROI was calculated by linear transformation of the CT value from a standard bone model to calculate the bone density parameters corresponding to the bone.

### 2.3 Immunohistochemistry and immunofluorescence

Tissue samples were fixed with 4% paraformaldehyde overnight and then transferred to an embedding cassette after ethylenediaminetetraacetic acid (EDTA) demineralization. The samples were dehydrated in gradient alcohol, cleared in xylene, immersed in paraffin, embedded, sectioned (5 μm) in a paraffin microtome, and placed in 60°C oven overnight; then, the sections were stored at room temperature. The sections were baked in a 60°C incubator overnight, followed by dewaxing with xylene, hydration, treatment with 3% hydrogen peroxide, antigen retrieval with sodium citrate solution, and blocking with goat serum for 1 h at room temperature. The sections were incubated (overnight at 4°C) with primary antibodies against IL-1β (1:200, company, country), MMP-9 (1:200, Affinity Biosciences, China), IL-10 (1:200, company, country), BMP-2 (1:200, company, country), and TNF-α (1:200, Bioss, China). Secondary antibody was added, and the sections were incubated at room temperature for 1 h. DAB staining, hematoxylin staining, dehydration, clearing, mounting, and image acquisition were performed. Image-Pro Plus software (Media Cybernetics, Inc., Rockville, MD, USA) was used to analyze the digitized images of the stained sections.

Immunofluorescence staining was performed using antibodies against CD68 (1:200, Boster, China), CD206 (1:200, Proteintech Group, USA), iNOS (1:200, Bioss, China), and cleaved caspase-1 (1:200, Affinity Biosciences, China). The number, location, and phenotype of macrophages and their relationship with NLRP3 inflammasomes in macrophages were evaluated. Nuclei were stained with DAPI. Stained sections were viewed under a confocal laser scanning microscope, and positive cell count analysis was performed using image J software.

### 2.4 ELISA

In accordance with the ELISA kit instructions, standard wells and sample wells were set up, and 50-μL samples of different concentration standards were added to the standard wells; and then, 50 μL was added to each sample well. Samples were not placed into blank wells. Except for the blank wells, 100 μL of horseradish peroxidase (HRP)-labeled antibody was added to each well, and the wells were sealed with a membrane and incubated at 37°C for 60 min. The liquid was discarded, and the wells were dried. After filling each well with washing solution, the plate was allowed to stand for 1 min, and then the washing solution was removed; the plate was washed five times. Fifty microliters each of substrates A and B was added to each well, and the plate was incubated at 37°C in the dark for 15 min. Then, 50 μL of termination solution was added, and the OD value at 450 nm was measured after 10 min. A standard curve was plotted, and the concentration of each sample was calculated using the equation for the standard curve. The concentration of the standard sample included 2000pg/ml, 1000 pg/ml, 500 pg/ml, 250 pg/ml, 125 pg/ml, 62.5 pg/ml, 31.25 pg/ml, 15.625 pg/ml.

### 2.5 RT−qPCR

Total RNA was extracted from tissue using RNAiso Plus (TaKaRa Biotechnology). The absorbance at 260 nm was measured using a NanoDrop 2000 spectrophotometer, and the RNA concentration was calculated. RNA purity was verified by the ratio of absorbance at 260 and 280 nm; a 260/280 nm absorbance ratio between 1.8 and 2.0 indicates good quality RNA for further experiments. RNA (2 µg) was reverse transcribed into cDNA using a First Strand cDNA Synthesis Kit (Thermo Scientific, USA). PCR was conducted using iTaq™ Universal SYBR^®^ Green (BioRad, USA), an Eco Real-time PCR system (Illumina, USA), and 1 µl of reverse-transcribed cDNA as a qPCR template. The reaction conditions were as follows: 95°C for 2 min; and 95°C for 5 s and 60°C for 30 s for 40 cycles. The 2−ΔΔCT relative quantification method was used, and GAPDH was used as the internal reference for analysis. The primer sequences are provided in **Table 1**.

**Table 1:** Primers for RT−qPCR.

Gene	Primers
GAPDH	5’- CCTGGAGAAACCTGCCAAGTATGAT -3’ AAGAATGGGAGTTGCTGTTGAAGTC
IL-1β	ATTCCGAGCCAAGAGAACATAG AGGCCACAGGGATTTTGTCG
NLRP3	CAGCGATCAACAGGCGAGAC AGAGATATCCCAGCAAACCTATCCA
BMP-2	TCACGAAGAAGCCATCGAG AGAAACTCATCAGTAGGGACAG
MMP-9	TGCAAAGTTGAACTCAGCC GTGTACACCCACATTTGCG

### 2.6 Statistical analysis

Each experiment was repeated three times, and all data are expressed as the mean ± standard deviation. SPSS software (version 25.0) was used for statistical analysis.

For *in vivo* animal experiments, the independent samples t-test were used to calculate the significance of differences. P<0.05 was considered a significant difference (P<0.05 *; P<0.01 **; and P<0.001 ***).

## 3 Results

### 3.1 Aging leads to reduced osteogenesis of bone substitute materials in aged rats

To study the differences in the osteogenic effects of bone substitute materials between young and aged rats *in vivo*, we constructed a cranial bone defect models in rats of different ages. We first observed the effects of Bio-Oss® in the induction of new bone tissue in young and aged rats in the middle stage of osteogenesis (6 weeks after surgery) and the late stage of osteogenesis (12 weeks after surgery). Micro-CT results indicated that there was significantly more new bone tissue in the defect area in young rats than in aged rats and that there were significant differences in BMD (**P<0.01) and BV/TV (**P<0.01). There was still a small number of gaps between the implanted materials in the defect areas of aged rats (**Figure 1A**). In addition, histological staining revealed that new bone formation in young rats included osteogenesis at the defect margins and the formation of bone islands around Bio-Oss® at the defect centers; in aged rats, there was mainly osteogenesis at the defect margins (**Figures 1B, C**). The difference in bone homeostasis in the early stage of osteogenesis between aged rats and young rats during the natural healing process was studied by observing the control groups. The local expression of BMP-2 was significantly lower in aged rats than in young rats (***P<0.001) (**Figures 1D, E**), whereas the local expression of MMP-9 was significantly higher in aged rats (*P<0.05) (**Figures 1F, G**). The above results indicate that the osteogenic induction ability of aging individuals was significantly reduced.

### 3.2 Significant differences in the local microenvironment of bone regeneration sites between young and aged rats

**Figure 2:**
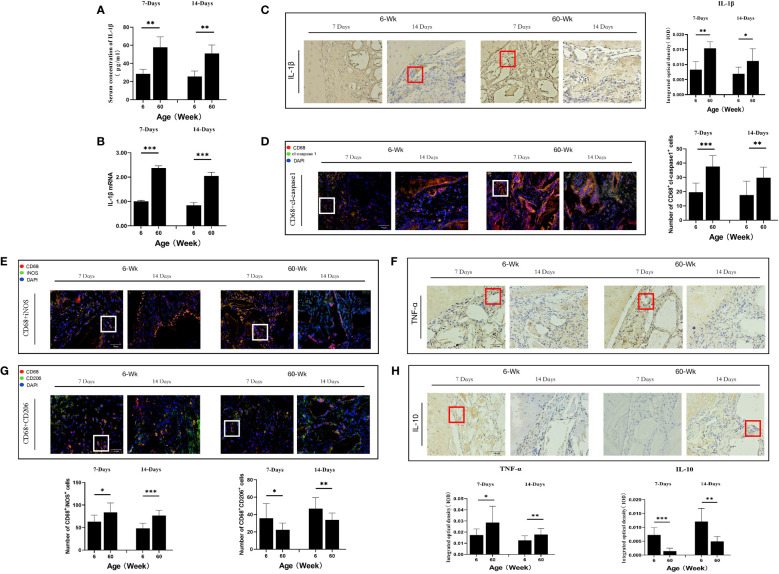
**(A)** ELISA determination of IL-1β levels in the serum of young and aged SD rats. **(B)** Local IL-1β gene expression in young and aged SD rats. **(C)** Immunohistochemical detection of IL-1β secretion in Bio-Oss® and its surrounding tissues on days 7 and 14 to assess differences in the local inflammatory microenvironment of defects in young and aged rats; Red square: positive cells. **(D)** CD68 (red) and cl-caspase-1 (green) co-immunolabeling to evaluate NLRP3 inflammasome expression in macrophages; nuclei were stained with DAPI; White square: double-positive cells. **(E, G)** CD68 (red), i-NOS (green), CD68 (red) and CD206 (green) co-immunolabeling was used to evaluate macrophage phenotypes; nuclei were stained with DAPI; White square: double-positive cells. **(F, H)** TNF-α and IL-10 were used to assess changes in the local inflammatory microenvironment; Red square: positive cells. Aged rats showed a significant increase in M1 macrophages and a significant decrease in M2 macrophages in the local microenvironment at the sites of bone defects, suggesting a delayed local inflammatory phase, elevated systemic and local IL-1β expression, and increased NLRP3 inflammasome activation in macrophages in aged rats. *P<0.05, **P<0.01, ***P<0.001.

The process of bone repair is biologically related to the host immune response. To investigate the correlation between the expression of NLRP3 inflammasomes and the potential of Bio-Oss® bone regeneration in the immunosenescent state, we examined changes in systemic and local NLRP3 inflammasome activation indicators. The serum IL-1β concentration in aged rats was higher than that in young rats (**P<0.01) (**Figure 2A**). In addition, compared with that in young rats, local IL-1β expression in the defect area was significantly increased in the aged rats (*P<0.05) (**Figures 2B, C**).

Macrophages are the main cell type that expresses cl-caspase-1 (25). Therefore, we analyzed the expression of cl-caspase-1 in macrophages at the local defect area by double immunofluorescence staining and found that the expression of cl-caspase-1 in local macrophages in aged rats was significantly higher than that in young rats (**P<0.01) (**Figure 2D**). In addition, the phenotype of macrophages in the local microenvironment of the defect was assessed. The number of local M1 macrophages was significantly higher in aged rats, and the local expression of TNF-α, an inflammatory factor secreted by M1 macrophages, was also increased (*P<0.05) (**Figures 2E, F**). In contrast, the number of M2 macrophages with anti-inflammatory repair functions was lower, and the expression of the anti-inflammatory factor IL-10 secreted by them was also reduced (*P<0.05) (**Figures 2G, H**). Therefore, under immunosenescence, the bone defect area was dominated by M1 macrophages with increased NLRP3 inflammasome activation. The resulting inflammatory microenvironment that increases IL-1β expression and thus promotes osteoclast differentiation may be lead to the poor bone regeneration potential of Bio-Oss® in aged rats.

### 3.3 The IL-4 immunomodulatory strategy promotes the osteogenesis of bone substitute materials in aged rats by regulating macrophage polarization

**Figure 3:**
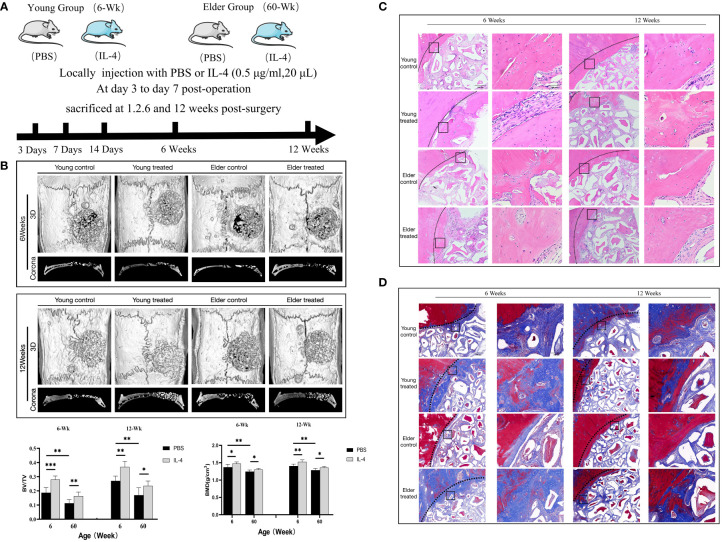
**(A)** Schematic of the animal research design. The effect of local immunomodulation via IL-4 on aged rats. **(B)** Three-dimensional and coronal micro-CT images of the cranial bone defect, BV/TV, and BMD at 6 and 12 weeks showing that the immunomodulatory effect of IL-4 promoted bone defect repair in young and aged rats, but the repair-promoting effect was weaker in the elderly treatment group than in the young treatment group **(C)** H&E and **(D)** Masson’s trichrome staining further confirmed the micro-CT results. *P<0.05, **P<0.01, ***P<0.001.

**Figure 4:**
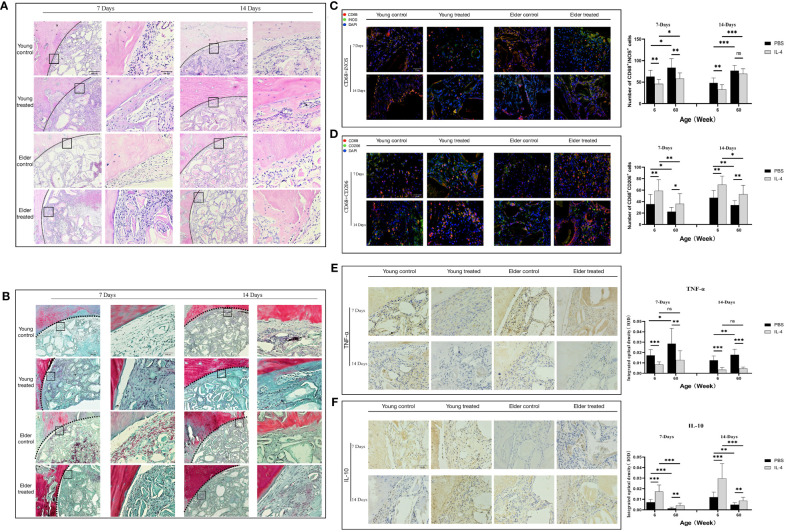
Local immunomodulation by IL-4 **(A)** H&E and **(B)** Masson’s trichrome staining. **(C, D)** CD68 (red), i-NOS (green), CD68 (red), and CD206 (green) co-immunolabeling was used to evaluate macrophage phenotypes; nuclei were stained with DAPI. **(E, F)** TNF-α and IL-10 were used to assess changes in the local inflammatory microenvironment. IL-4 recruited more macrophages at the sites of bone defects in aged rats and altered the M1/M2 ratio to form a promoted healing microenvironment by promoting the polarization of M1-type macrophages to M2-type macrophages. *P<0.05, **P<0.01, ***P<0.001. ns, no significance.

To study the effect of the regulation of macrophage polarization on the bone regeneration potential of Bio-Oss® under immunosenescent conditions, IL-4 was locally injected at the Bio-Oss® implantation site in young and aged rats (3-7 days after surgery) (**Figure 3A**). Micro-CT 3D reconstruction (**Figure 3B**) indicated the new bone tissue in the elderly treatment group was significantly increased compared to that in the elderly control group. There was more new bone tissue at the defect edges in the elderly treatment group than in the elderly control group. Additionally, a small amount of bone islands formed around Bio-Oss® at the defect centers in the elderly treatment group (**Figures 3C, D**). Regarding the early bone defect area, the application of IL-4 caused the local recruitment of more cells and accelerated the formation of new bone at the bone defect edges (**Figures 4A, B**).

The phenotype of macrophages in the local microenvironment of the defect was analyzed. IL-4 treatment reduced the number of M1 macrophages in the elderly treatment group compared with the elderly control group at 7 days after surgery (**P<0.01) (**Figure 4C**). In addition, the reduced expression of the local inflammatory factor TNF-α (**P<0.01) (**Figure 4E**). And the increases in the number of M2 macrophages (*P<0.05), anti-inflammatory factor IL-10 expression (**P<0.01) (**Figures 4D, F**). The local M1/M2 ratio indicated the formation of a microenvironment that promoted healing, consistent with the trend of changes in the young treatment group. However, at 14 days after surgery, there was a difference between the elderly treatment group and the young treatment group. The local microenvironment status of the young treatment group remained the same as that at 7 days after surgery. While the number of local M1 macrophages increased in the elderly treatment group compared with 7 days after surgery (*P<0.05), resulting in changes in the M1/M2 ratio and local microenvironment. These results suggest that in aged rats, IL-4 also promotes Bio-Oss®-induced bone regeneration by regulating macrophage polarization; however, the effect seems to gradually decrease due to the overall chronic low-intensity inflammatory state.

### 3.4 IL-4 inhibits NLRP3 inflammasome activation and downregulates IL-1β expression during early bone regeneration in aged rats

**Figure 5:**
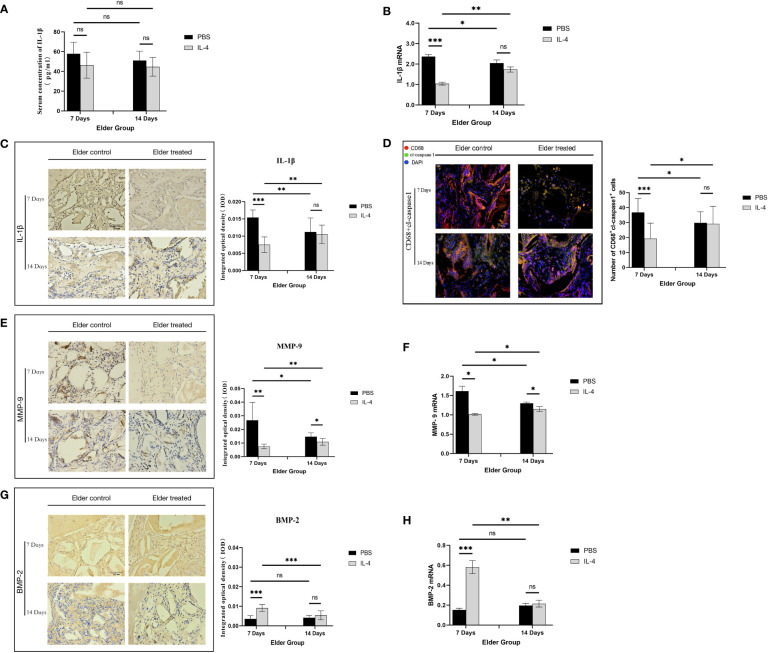
Local immunomodulation by IL-4 **(A)** ELISA detection of IL-1β in the serum of aged SD rats. **(B)** Changes in local IL-1β gene expression in aged SD rats. **(C)** Immunohistochemical detection of IL-1β secretion in Bio-Oss® and its surrounding tissues on days 7 and 14 to assess differences in the local inflammatory microenvironment of defects in aged rats. **(D)** CD68 (red) and cl-caspase-1 (green) co-immunolabeling was used to evaluate NLRP3 inflammasome expression in macrophages; nuclei were stained with DAPI. **(E, G)** Immunohistochemical detection of BMP-2 and MMP-9 secretion in Bio-Oss® and its surrounding tissues on day 7 and day 14. **(F, H)** Local BMP-2 and MMP-9 gene expression in aged SD rats was used to assess the dynamic balance of local bone regeneration and bone resorption in the defects of aged rats. Local immunomodulation in aged rats resulted in reduced M1 numbers, inhibition of NLRP3 inflammasome activation, downregulation of IL-1β expression, reduced osteoclast fractionation, changes in the M1/M2 ratio, and increased BMP-2 expression, thereby promoting bone regeneration. *P<0.05, **P<0.01, ***P<0.001. ns, no significance.

The regulatory mechanism by which IL-4 promotes Bio-Oss®-induced bone regeneration in aged rats was further explored. First, ELISA detection of systemic IL-1β serum concentrations in the aged rat group indicated that was no significant difference between the treated and control groups (P>0.05) (**Figure 5A**), suggesting that the local immunomodulatory effect of IL-4 has no effect on the expression of systemic NLRP3 inflammasomes. Local IL-1β expression at 7 days after surgery was significantly lower in the elderly treatment group than in the elderly control group (***P<0.001). In contrast, at 14 days after surgery, there was no significant difference between the two groups (P>0.05), and an increase in IL-1β expression in the elderly treatment group was observed (**P<0.01) (**Figures 5B, C**). The expression of cl-caspase-1 also showed the same trend (**Figure 5D**).

The expression of MMP-9 decreased in aged rats treated with IL-4 compared with the control group (*P<0.05), but an increase was observed at 14 days after surgery compared with 7 days (*P<0.05), consistent with the trend of IL-1β expression (**Figures 5E, F**). This result demonstrates that the IL-4 immunomodulatory strategy inhibits local NLRP3 inflammasome activation and IL-1β secretion in early osteogenesis in aged rats, thereby inhibiting osteoclast differentiation. BMP-2 expression increased in the treatment group compared with the control group (***P<0.001), but was not significantly different from that in the control group at day 14 after surgery (P>0.05). At 14 days after surgery, BMP-2 expression was lower than 7 days in the treatment group (**P<0.01) (**Figures 5G, H**). This result demonstrated that after the IL-4 intervention ended, the immunomodulatory effect gradually diminished and the M1/M2 local microenvironment changed, thereby suppressing BMP-2 expression.

## 4 Discussion

Aging causes major alterations in the immune system and thus results in the increased susceptibility to disorders. It is not clear whether immunomodulatory strategies commonly used in the general population are effective in the elderly. In this study, the expression of local NLRP3 inflammasomes increased during immunosenescence, potentially affecting the osteogenic function of Bio-Oss®. Local application of IL-4 stimulated the polarization of macrophages from the M1 to M2 phenotype, thereby increasing the expression of the osteogenesis-related factor BMP-2 in early osteogenesis by increasing the number of M2 macrophages to achieve an ideal M1/M2 ratio, resulting in the formation of a local microenvironment conducive to healing and promoting the osteogenic effect of Bio-Oss®. The improved local inflammatory microenvironment inhibited NLRP3 inflammasome activation and reduced the expression of the osteoclast-associated factor MMP-9. However, due to the chronic low-intensity inflammatory state of the whole body in aged rats, the local inflammatory microenvironment state gradually recovered after the IL-4 intervention was stopped, and the immunomodulatory effect of IL-4 gradually diminished.

Currently, more research is focused on how to promote fracture healing in elderly individuals, including local injections of drugs to control inflammation and hematopoietic cell engraftment (27, 28). However, fewer studies on bone substitute materials mediating bone regeneration in elderly individuals are available. In the process of bone tissue regeneration induced by bone substitute materials, the molecular events that control bone tissue regeneration involve a complex signaling network, including tissue damage, cell death, cell recruitment, cell proliferation, cell differentiation, and tissue formation (29). Studies have found that the immune system and the skeletal system are closely related and that the two systems share many cytokines, receptors, signaling molecules, and transcription factors, which are together referred to as osteoimmunology (30, 31). Immune cells play a key role in bone homeostasis. When an implant is recognized by the immune system, an immune response is triggered, which affects the biological behavior of bone cells (32). This immune response may ultimately determine the fate of bone biomaterials in the body (33). Local regulation of the host immune response through cytokine delivery to improve bone substitute material-mediated new bone formation and angiogenesis is considered a promising therapeutic strategy (13, 34).

The proper sequence of inflammatory signals during bone regeneration and the subsequent anti-inflammatory signals are critical for normal bone healing (35). For example, when TNF-α is applied to the fracture site of mice immediately after bone injury, the healing of the fracture can be accelerated; however, continuous application of TNF-α to the fracture site hinders the healing process (36). Although studies have supported that the polarization of macrophages into the M2 type is conducive to bone formation (37), some studies have demonstrated that hyperpolarization can also induce fibrosis and promote the fusion of macrophages into foreign body giant cells *in vivo* and *in vitro* (38). Therefore, the precise regulation of macrophage polarization is crucial (39). *In vitro* studies have shown that after 72 h of interaction between rat osteoblastic MC3T3 cells and M1 macrophages, IL-4 regulation enhanced the osteogenic capacity, promoted ALP activity, increased OCN expression, and enhanced bone tissue mineralization (12). Therefore, in this experiment, we locally injected IL-4 into bone defects on postoperative day 3 as an immunomodulator of the ideal sequence of changes in inflammatory signals and anti-inflammatory signals. The experimental results indicate that such a signaling sequence is equally important in aged rats, where IL-4 intervention shortens the inflammatory phase of the local microenvironment and promotes Bio-Oss®-mediated osteogenic effects. At the same time, macrophage aging may affect the different stages of bone repair, resulting in age-related insufficient bone regeneration. Clark et al. (40) found that the number of infiltrating macrophages in the callus of aged and young mice was similar but that the expression of M1/proinflammatory genes was upregulated in the macrophages of aged mice, and the macrophages showed a stronger proinflammatory and M1 phenotype. In this study, analysis of the local macrophage phenotype also revealed that there were more M1 macrophages in the local defect of aged rats.

In addition, we also observed that NLRP3 inflammasome activation mainly occurred in M1 macrophages. As we all know, IL-4 acts as an immunomodulatory factor to suppress inflammatory responses (41). IL-4 significantly inhibited NLRP3 inflammasome assembly in response to lipopolysaccharide (LPS) or LPS/ATP stimulation and even attenuated NLRP3 inflammasome activation in reconstituted NLRP3-expressing macrophages (42). Mechanically, IL-4 binds to the IL-4 receptor and activates the signal transducer and activator of transcription 6 (STAT6) signal transduction pathways as well as peroxisome proliferator activated receptor-γ (PPARγ), thus inhibiting nuclear factor-kB (NF-kB)-related inflammatory responses. Notably, the assembly and activation of the NLRP3 inflammasome is highly associated with the transcription factor NF-kB (43, 44). Nevertheless, the negative regulatory effect of IL-4 on NLRP3 inflammasome is not dependent on the inhibition of mRNA or protein expression and pro-inflammatory cytokine expression. Supporting above observation, IL-4 can inhibit inflammasome activation even in NLRP3 recombinant macrophages, and thus IL-4-mediated NLRP3 inflammasome inhibition is independent of TLR-NF-κB signal transduction as well as STAT6 transcription and mitochondrial ROS (42). In this experiment, when IL-4 was applied to aged rats for comparison against young rats, it promoted the polarization of M1 macrophages to the M2 macrophages and was able to alter the inflammatory microenvironment in aged rats by reducing NLRP3 inflammasome activation. Interestingly, after cessation of the IL-4 intervention, local NLRP3 inflammasome activation was increased in aged rats, which we suspect is due to altered macrophage function in aging individuals and inflammation-related pathways upregulation in plasma (16, 19, 40, 45). With the continuous recruitment of M1 macrophages to the defect area, NLRP3 inflammasomes were continuously activated, resulting in changes in the local immune microenvironment again. In accordance with above findings, in our study, IL-4 was demonstrated to inhibit the activation of NLRP3 inflammasome and downregulate the expression of IL-1β in macrophages, suggesting that IL-4 may play a role in regulating inflammatory response by regulating NLRP3 inflammasome. However, whether IL-4 acts directly on NLRP3 inflammasomes or indirectly through macrophages on NLRP3 inflammasomes requires further validation.

This experiment has some limitations. First, in this study, we only analyzed osteogenesis after the application of IL-4 in aged rats and did not explore the cause of this mechanism at the cellular pathway level, which should be addressed in future studies. Second, regarding the method of drug administration, local injection for five consecutive days could aggravate the local inflammatory response in the defect area, and therefore, other drug administration methods, such as loading on sustained-release materials, should be tested. In addition, the local microenvironment was changed again after cessation the administration of IL-4 to aged rats. Therefore, it may be necessary to increase the concentration of IL-4 and the duration of continuous application, and approach that should be tested in future studies.

In summary, this study demonstrated that the macrophages at the site of the bone defect after implantation of bone substitute material in immunosenescence mainly present an M1 phenotype, with increased NLRP3 inflammasome activation that changes the local microenvironment, resulting in a poor osteogenic effect of Bio-Oss® in aging rats. The exogenous use of IL-4 inhibited local NLRP3 inflammasome activation and downregulated IL-1β expression by regulating macrophage polarization in early osteogenesis in aged rats, significantly improving the local inflammatory microenvironment under immunosenescence and inhibiting osteoclast differentiation to promote bone regeneration. However, this immunomodulatory effect gradually diminished. Implying that the immunomodulatory strategy of regulating macrophage polarization through IL-4 may be a promising strategy for promoting bone regeneration induced by bone substitute materials *in vivo* in elderly individuals, further studies are needed in the future to clarify its safety and side effects.

## Data availability statement

The original contributions presented in the study are included in the article/**Supplementary Materials**, further inquiries can be directed to the corresponding author/s.

## Ethics statement

The animal study was reviewed and approved by the Ethics Committee of Guizhou Medical University.

## Author contributions

Designed the research studies: AT, ZT, and DL. Conducted the experiments: DL, XL, JZ, and ZT. Acquired the data: DL, XL, and JZ. Analyzed the data: AT, DL, XL, and ZT. Wrote the manuscript: AT, ZT, and DL. Manuscript revision: AT. All authors contributed to the article and approved the submitted version.

## Supplementary material

Supplementary Material
**(A)** Surgical modeling. **(B)** CD68 (red) and i-NOS (green) co-immunolabeling. **(C)** CD68 (red) and CD206 (green) co-immunolabeling. **(D)** CD68 (red) and cl-caspase-1 (green) co-immunolabeling.
